# Modelling the effects of past and future climate on the risk of bluetongue emergence in Europe

**DOI:** 10.1098/rsif.2011.0255

**Published:** 2011-06-22

**Authors:** Helene Guis, Cyril Caminade, Carlos Calvete, Andrew P. Morse, Annelise Tran, Matthew Baylis

**Affiliations:** 1 Liverpool University Climate and Infectious Diseases of Animals (Lucinda) Group, Faculty of Veterinary Science, Leahurst Campus, University of Liverpool, Neston CH64 7TE, UK; 2 CIRAD, UPR AGIRS, Montpellier F-34398, France; 3 CIRAD, UMR CMAEE, Montpellier F-34398, France; 4 School of Environmental Sciences, University of Liverpool, Liverpool L69 7ZT, UK; 5 Unidad de Sanidad y Producción Animal, Centro de Investigación y Tecnología Agroalimentaria (CITA), Gobierno de Aragón, Zaragoza, Spain; 6 CIRAD, UMR TETIS, F-34093 Montpellier, France

**Keywords:** climate change, vector-borne disease transmission, basic reproductive ratio, emergence, bluetongue, *Culicoides*

## Abstract

Vector-borne diseases are among those most sensitive to climate because the ecology of vectors and the development rate of pathogens within them are highly dependent on environmental conditions. Bluetongue (BT), a recently emerged arboviral disease of ruminants in Europe, is often cited as an illustration of climate's impact on disease emergence, although no study has yet tested this association. Here, we develop a framework to quantitatively evaluate the effects of climate on BT's emergence in Europe by integrating high-resolution climate observations and model simulations within a mechanistic model of BT transmission risk. We demonstrate that a climate-driven model explains, in both space and time, many aspects of BT's recent emergence and spread, including the 2006 BT outbreak in northwest Europe which occurred in the year of highest projected risk since at least 1960. Furthermore, the model provides mechanistic insight into BT's emergence, suggesting that the drivers of emergence across Europe differ between the South and the North. Driven by simulated future climate from an ensemble of 11 regional climate models, the model projects increase in the future risk of BT emergence across most of Europe with uncertainty in rate but not in trend. The framework described here is adaptable and applicable to other diseases, where the link between climate and disease transmission risk can be quantified, permitting the evaluation of scale and uncertainty in climate change's impact on the future of such diseases.

## 1. Introduction

Climate change may cause vector-borne diseases to shift in distribution because the vectors' ecology and the pathogen development rate within them strongly depend on environmental conditions. In some cases, shifts to previously unexposed populations of humans and animals could have severe or even devastating consequences. Modelled projections of how vector-borne diseases will respond to climate change are needed so that measures of mitigation or adaptation can be taken.

Bluetongue (BT), a viral disease of ruminants transmitted by biting midges (*Culicoides* spp.), is considered by many to represent one of the most plausible examples of climate change driving the emergence of a vector-borne disease [1]. BT is widely distributed in Africa, Asia, Australia, South America and North America. In Europe, although a few sporadic outbreaks occurred in the last century, BT had never established itself in the long term. A dramatic change occurred in 1998 when an unprecedented series of outbreaks began [2], involving nine different serotypes of the virus, causing the deaths of millions of ruminants, and major economic consequences for the region. In Europe, over 110 000 outbreaks were declared to the World Animal Health Organization (OIE) between 1998 and 2010; over 80 000 of these were owing to BT virus serotype 8. The emergence of the disease in southern Europe has been attributed, in part, to the northwards spread of the afro-tropical vector, *Culicoides imicola*, across the Mediterranean basin [3]. This species is currently absent from northern Europe. However, BT occurred for the first time in northwest (NW) Europe in 2006, transmitted by indigenous species of *Culicoides*, most importantly members of the Obsoletus complex [4,5].

Possible causes of BT's emergence in Europe have been discussed by Purse *et al*. [3] (see the electronic supplementary material for detailed discussion on possible non-climatic drivers). They conclude that it seems improbable that biotic or non-climatic abiotic factors could have been responsible for this emergence. On the contrary, evidence is presented that the emergence of BT in southern Europe occurred in the same place and at the same time as regional warming between the 1980s and 1990s, thereby providing support for a role of climate change [3]. However, this correlation does not quantify precisely the link between climate and disease transmission parameters to explain the observed emergence. Thus, although it suggests possible mechanisms by which warming could have affected the disease, it does not identify which mechanisms are the most important. Additionally, it does not account for the emergence of BT in northern Europe in 2006. Nevertheless, this link with climate change, and the recent spread of disease in Europe, makes BT an excellent example for developing models of how climate change will influence diseases in the future, with the opportunity to validate models against the observed past outbreak occurrence.

In this paper, we present a framework for the evaluation of the effects of past and future climate on the risk of emergence of BT. The approach is based on an epidemiological model of disease transmission, the basic reproduction ratio, *R*_0_, adapted for a two-host and two-vector disease. Four parameters of the model are known to be climate-driven. For three parameters, the equations linking the parameter to temperature have been described in the literature. To estimate the link of the fourth parameter with climate, new vector abundance models were developed. By integrating the observed high spatial resolution (25 km) climate data in the disease transmission model, the disease transmission risk can be evaluated for past periods of time. This allows us to evaluate whether the integrated climate-driven model successfully reproduces aspects of BT's past observed occurrence, including the distribution of its vectors and the emergence of the disease in NW Europe in 2006. Examining whether past epidemiological events have been driven by climate is essential before projecting models in the future [6]. In order to drive the model into the future, simulations of future climate are then integrated into the *R*_0_ model. Two steps are necessary for this. First, the model projections for past climate, obtained by integrating an ensemble of 11 regional climate models (RCMs), are compared with the *R*_0_ model driven by the observed climate data to make sure that the ensemble of climate models is able to reproduce past patterns of BT. In a second step, the *R*_0_ model is then driven by the simulations of the ensemble of 11 RCMs under a climate change scenario to evaluate the transmission risk for a future time period up to 2050. Using an ensemble of models instead of a single model, it is important to take into account the uncertainties related to the climate simulations which affect the transmission simulations.

## 2. Material and methods

### 2.1. Evaluating the basic reproduction number *R*_0_ for bluetongue

The basic reproduction ratio (*R*_0_, the number of secondary cases arising from the introduction of one infected host in a susceptible population) is a powerful tool to assess the risk of disease transmission in the event of viral introduction [7] (at the onset of the epidemic). Gubbins *et al.* [8] and Hartemink *et al.* [9] have modelled BT's *R*_0_ as a two-host disease, as cattle and sheep play different epidemiological roles [8,9]. Indeed, cattle, as opposed to sheep, are usually not clinically affected [10,11] and harbour a longer viraemia [12]. *R*_0_ for BT was adapted from Gubbins *et al.* [8]
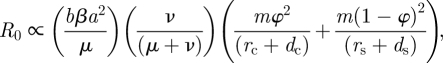
where *b* is the probability of transmission from vector to host, *β* the probability of transmission from host to vector, *a* the biting rate, *μ* the vector mortality rate, 1/*ν* the mean extrinsic incubation period, *m* the vector-to-host ratio, *φ* the proportion of bites on cattle, 1/*r* the duration of viraemia (in cattle *r*_c_ and in sheep *r*_s_) and 1/*d* the disease-induced mortality rate (in cattle *d*_c_ and in sheep *d*_s_).

The formula developed by Gubbins *et al.* [8] is very similar to the one derived separately by Hartemink *et al.* [9] and used to model *R*_0_ for BT across The Netherlands. Gubbins *et al.* [8] distinguished a vector-to-cattle ratio from a vector-to-sheep ratio in their formulae. Here, we propose a method that inherently estimates these two parameters together. However, we considered that this ratio had to be computed distinctively for *C. imicola* and for the Obsoletus complex because of the dissimilar distributions of these species (see the estimation of *m*).

We chose to only consider sheep and cattle as BT hosts. We did not consider other domestic hosts such as goats because of their small population sizes in Europe. We did not consider wild hosts because very little information is available to include them as a third host (no parameter estimates are available for wildlife) and because their role seems to be more important in the persistence of the disease than at the onset of the disease (see electronic supplementary material).

Although transplacental transmission in hosts has been shown to occur in cattle with BT serotype 8 virus [13], and probably represents an important overwintering mechanism, it has been considered to be insignificant at the onset of the epidemic [9].

Four parameters were considered to be constant in space and time: the probabilities of transmission from vector to host (*b*) and from host to vector (*β*), the duration of viraemia (1/*r*) and the disease-induced mortality rates (1/*d*) (estimates of all parameters are given in table 1). As host preferences of the two European vectors are not well described, in our model, the proportion of bites on cattle, *φ*, and on sheep, (1−*φ*), is defined as the proportion of cattle and sheep available, respectively. The availability of cattle and sheep was based on gridded estimates of livestock density in 2005 [25].

**Table 1.** *R*_0_ parameters for the two-host bluetongue transmission model. *T*, temperature.

parameter	definition	estimation or range (value chosen)	reference
*b*	probability of transmission from vector to host	0.8–1.0 (0.9)	[14]
*β*	probability of transmission from host to vector	0.001–0.15 (0.075)	[15–17]
*a*	biting rate (d^−1^)	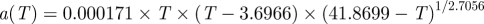	[18,19]
*μ*	vector mortality rate; (d^−1^)	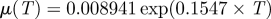	[20]
1/*ν*	mean extrinsic incubation period (day)	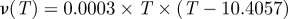	[18]
*m*	vector-to-host ratio	see main text	
*φ*	proportion of bites on cattle	number of cattle/number of sheep and cattle	
1/*r* (*r*_c_, *r*_s_)	duration of viraemia (cattle, sheep) (day)	cattle: 20.6; sheep: 16.4	[21–23]
1/*d* (*d*_c_, *d*_s_)	disease-induced mortality rate (cattle, sheep); d^−1^)	cattle: 0 sheep: 0.001–0.01 (0.005)	[11,24]

Four parameters—*a*, the vector biting rate (the daily probability of a midge feeding on a susceptible host); *μ*, the vector mortality rate; *ν*, the reciprocal of viral extrinsic incubation period (the time taken for a midge to become infectious after taking an infected blood meal); and *m*, the vector-to-host ratio—are known to exhibit strong climatic dependence [8,26] and therefore vary in space and time (table 1). Equations linking the first three parameters to temperature for North American midges were obtained from published literature (table 1 and electronic supplementary material for more details on how the parameters were estimated). The relationships with temperature are shown in electronic supplementary material, figure S1.

Estimation of the vector-to-host ratio (*m*) is complex. Indeed, *Culicoides* are usually sampled in the field using light traps; however, there is no established method of converting trap catch data to population size. Here, we assume that a trap acts like a host and attracts a number of midges proportional to what a host would attract. Under this assumption, a catch does not directly estimate the vector population size but, instead, is an estimate of the vector-to-host ratio. In other words, the number of vectors caught in a trap depends on both the population size of the vectors, and the number of hosts (and traps) to which they are attracted. This assumption seems more realistic as *R*_0_ is then proportional to trap catches, whereas under the assumption that trap catches reflect a given percentage of the vector population size, *R*_0_ may be very high in areas where there are a few vectors but very low host densities (such as cities for example).

Furthermore, the vector-to-host ratio has to be estimated separately for the two vectors, *C. imicola* and the Obsoletus complex, as they have dissimilar ecologies and distributions [27,28]. Statistical models of trap catches for *C. imicola* and Obsoletus complex have been published in recent years [26,29–32], but all include variables that cannot be projected under future climate change scenarios, such as the normalized difference vegetation index. We therefore developed new vector distribution models restricting explanatory variables to ones which could be projected in the future.

**Figure 1.**
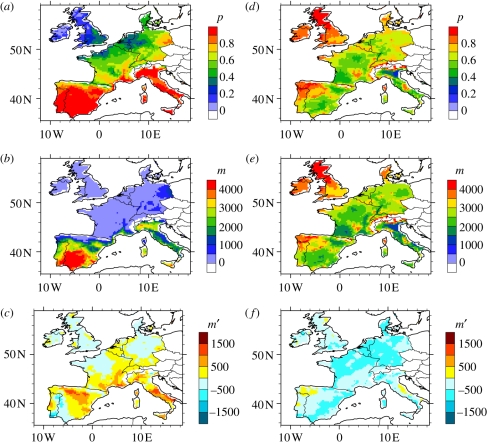
Mean probability of presence, vector-to-host ratio, and anomalies for *C. imicola* and the Obsoletus complex. (*a*) Mean probability of presence for *C. imicola* (August–September–October (ASO) 1961–1999). (*b*) Vector-to-host ratio for *C. imicola*. (*c*) Vector-to-host ratio anomaly for *C. imicola* for 2000–2008 (relative to the 1961–1999 mean). (*d*) Mean probability of presence for the Obsoletus complex (ASO 1961–1999). (*e*) Vector-to-host ratio for the Obsoletus complex. (*f*) Vector-to-host ratio anomaly for the Obsoletus complex for 2000–2008 (relative to the 1961–1999 mean).

Using data provided by the Spanish BT national surveillance programme on *Culicoides* trap catches set in livestock holdings from 2004 to 2006, presence–absence and abundance models were developed following the methods of Calvete *et al*. [26]. At each farm, the maximum catch per year was considered to be the best estimate of midge abundance. Indeed, trap catches can be extremely variable throughout the year and even between two consecutive nights, and highly dependent on local meteorological conditions [33]. Maximum catches are classically considered to be the best approximation of the midge abundance as any smaller catches could be underestimations owing to meteorological conditions being temporarily not favourable [33]. The dataset was divided into a training (*n* = 330) and an independent validation (*n* = 255) dataset. Using an information-theoretic paradigm based on Akaike's criterion, the best logistic regression models of the probability of occurrence of each vector were selected (electronic supplementary material, table S1). Five bioclimatic variables were included in the models: mean annual temperature, annual precipitation, their variation coefficients (all extracted from a 1950–2000 monthly climatic series) and a sun index, derived from a digital elevation model. Both occurrence models have a fairly good discriminatory capacity in internal (the area under the receiver-operating characteristic curve is 0.811 for *C. imicola* and 0.736 for the Obsoletus complex) and external (0.779 for *C. imicola* and 0.710 for the Obsoletus complex) validation. Midge abundance was obtained by fitting a regression equation to the predicted probability of detection (electronic supplementary material, table S2). The vector-to-host ratio was obtained by calibrating the abundance of each vector on a 0–5000 scale [8] (figure 1*b*,*e*) with areas of maximal abundance (obtained for a probability of occurrence equal to 1) having a ratio of 5000. The ratios calculated for the two vectors were then summed to compute *m* for all vectors.

Contrary to others [9], we considered that *R*_0_ for BT could not be quantified exactly because of the lack of knowledge on specific estimates of some of the *R*_0_ parameters for the European species of vectors (such as *a*, *μ* and *ν*). Therefore, we conservatively present *R*_0_ anomalies, the change in *R*_0_ in one time period relative to a baseline.

### 2.2. Climate data

Temporal variation in *R*_0_ was derived from three sources of climate data: a high-resolution, observed climate dataset for 1961–2008; and two ensembles of climate model simulations provided by the ENSEMBLES research theme 3 (available at http://ensemblesrt3.dmi.dk/). Observed temperature and rainfall are estimated from the E-OBS gridded dataset (25 km resolution) which is derived through the interpolation of station measurements [34].

Regional scenarios for climate change impacts assessment require finer spatial scales than those provided by general circulation models (GCMs), which have a coarse resolution (about 300 km). The ENSEMBLES European project (http://ensembles-eu.metoffice.com/) provides improved RCMs, at spatial scales of 25 km, for both recent past (1961–2000) and future climate scenarios (1950–2050). Models covering the European domain with a regular 0.25° step consistent with the observation grid were retained. Two ensembles of simulations are considered, the control experiments (SimCTL) and the scenario experiment (SimA1B).

In the SimCTL experiment (1961–2000), all RCMs are forced at their boundaries by the ERA40 reanalysis (the ‘best guess’ of the observations that uses both modelling and different sources of observations through data assimilation) [35]. Observed external forcing (greenhouse gases, solar, volcanic, aerosols) is applied to all RCMs.

In the SimA1B experiment (1961–2050), the RCMs are forced at their boundaries by a GCM with a coarser resolution (about 300 km) forced by the A1B emission scenario (median scenario in terms of CO_2_ emissions) [36]. Different GCMs are used to drive the RCMs. This explains, in part, the large spread in the different scenarios with respect to the control run (in which all RCMs have the same boundary conditions, namely they are all driven by the ERA40 reanalysis). As each RCM realization is based on a different model (with different physical parametrization), and as the GCM which provides the RCM boundary conditions vary from one RCM to another, we can assume that the various RCM projections are independent of one another.

The 11 selected RCMs (and operational centre which developed them) are: C4IRCA3 (Met Éireann, Ireland), CNRM-RM4.5 (CNRM, Météo-France), DMI-HIRAM5 (DMI, Denmark), ETHZ-CLM (ETHZ, Switzerland), ICTP-RegCM3 (ICTP, Italy), KNMI-RACMO2 (KNMI, Netherlands), METO-HC (Met Office, UK), MPI-M-REMO (MPI, Germany), OURANOSMRCC (OURANOS, Canada), SMHIRCA (SMHI, Sweden) and UCLM-PROMES (UCLM, Spain).

RCM-simulated precipitation and temperature were bias-corrected on a monthly mean basis with respect to the E-OBS observed dataset over the 1961–1999 reference period (see the electronic supplementary material).

### 2.3. Integration of climate data in the transmission model

We acknowledge that the entomological data used to build the models covered only a restricted geographical area (Iberia). Thus, model projections were limited to Western Europe and results of midge distribution were carefully examined (see §3) before computing *R*_0_ anomaly maps. For some outputs, a distinction was made between southwest (SW) Europe (below the northern border of Spain, i.e. the area where *C. imicola* is abundant) and NW Europe (above Switzerland, where *C. imicola* is absent).

In Europe, BT is highly seasonal and occurs mostly in late summer to autumn. For each climate dataset, the temperature-dependent parameters (*a, *μ*, *ν**) included in the *R*_0_ model are computed for August, September and October (ASO) and then averaged to build a seasonal mean for each year. The vector-to-host ratio (*m*) is computed on an annual basis, and then integrated with the seasonal mean of the other parameters to compute *R*_0_ for ASO. For each of the two ensembles (SimCTL and SimA1B), the ensemble mean is then estimated by averaging the *R*_0_ values for each individual RCM simulation. These estimates were averaged to give long-term and decadal means. To evaluate the specific effect of each parameter on *R*_0_ anomalies (figure 3), all parameters but one were held constant. To investigate the relative influences of change in temperature versus rainfall from the SimA1B ensemble on future change in *R*_0_ anomaly, each was held constant (temperatures at 20°C and 25°C, rainfall at 250 and 500 mm) in turn, while the other was allowed to vary (figure 6). Changes in temperature and rainfall over the 1960–2050 period are shown in the electronic supplementary material, figure S3. For a given ensemble, the spread of simulated *R*_0_ (figure 7) is defined as 1 s.d. of all RCM projections with respect to the ensemble mean. The multi-model sign consistency is computed assigning +1 (−1) to each RCM projection if an increase (decrease) in *R*_0_ is simulated. This is averaged and multiplied by 100 to display percentages.

## 3. Results

### 3.1. Modelled vector-to-host ratios

Modelled vector-to-host ratios for *C. imicola* for the 1961–1999 period (figure 1*b*), driven by the observed climate data, reproduce the past situation in Spain and southern Portugal, the only parts of SW Europe in which this species was known to occur before 1998. The anomaly for the period 2000–2008 (i.e. the change in modelled ratio during this time period compared with that of 1961–1999) reproduces remarkably well the recent spread of *C. imicola* to areas of northern Spain and southeast France (figure 1*c*). The precise time of introduction is not known, but *C. imicola* was detected for the first time in 2000 in Corsica [37], in southeast France in 2003 [38] and in northern Spain in 2007 [39]. The species is known to be spreading in these areas. However, the model does not reproduce its first detection in Catalonia in 2002 [40]. In Italy, the situation is not as clear: *C. imicola* was first detected in 2000 [41] but entomological surveillance was unable to detect a range of expansion between 2002 and 2007 [42]. The model over-predicts the presence of *C. imicola* in north Italy, where it has not yet been detected.

Regional data on the distribution of the indigenous Obsoletus complex do not yet exist. Nevertheless, modelled vector-to-host ratios for the Obsoletus complex for the 1961–1999 period confirm its known, very widespread distribution (figure 1*e*). Negative anomalies of the Obsoletus complex for 2000–2008 occur across almost the entire region suggesting that, recently, climate has caused the density of this complex to decrease (figure 1*f*).

### 3.2. Past *R*_0_ anomalies

**Figure 2.**
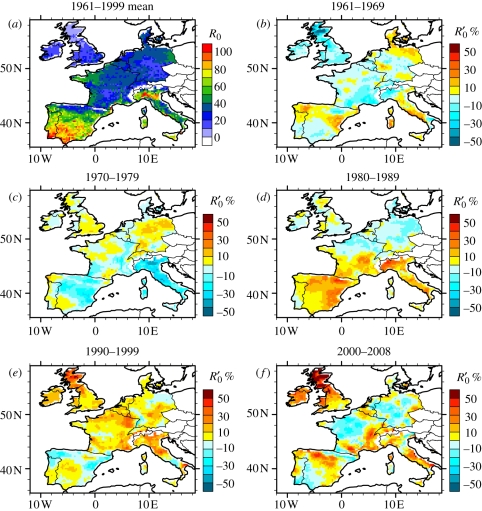
Long-term mean and modelled *R*_0_ decadal variability. (*a*) Long-term mean *R*_0_ for the ASO season (the average is computed for the 1961–1999 period). The magnitude has been scaled to vary between an arbitrary range between 0 and 100%. (*b*–*f*) *R*_0_ relative anomalies (%) with respect to the reference mean (1961–1999) for different decades. *R*_0_ is estimated from the observed climate dataset.

The simulated mean *R*_0_ (figure 2*a*), again based on observed climate data, depicts an increasing North–South gradient and correctly identifies southern Spain and Portugal as key areas at risk of BT for the 1961–1999 period. Although no BT occurred in north Italy during that period (see OIE Handistatus II Annual animal disease status Europe/2002/Bluetongue Animal health status at http://www.oie.int/hs2/sit_mald_cont.asp?c_mald=10&c_cont=4&annee=2002), the model projects high risk of disease transmission in the event of viral introduction. In the 1960s and 1970s (figure 2*b*,*c*), most areas had negative *R*_0_ anomalies and hence low risk of disease transmission (in the event of viral introduction) relative to the 1961–1999 mean. In the 1980s (figure 2*d*), areas of Spain, southern France and NW Italy displayed positive *R*_0_ anomalies, suggesting an increase in the risk of disease transmission in the event of viral introduction. In the 1990s and early 2000s, strong positive *R*_0_ anomalies in NW Europe including the UK are highlighted (figure 2*e*,*f*). The climate conditions over NW Europe could thus have been favourable to BT transmission for 15 years before the virus was introduced in 2006.

**Figure 3.**
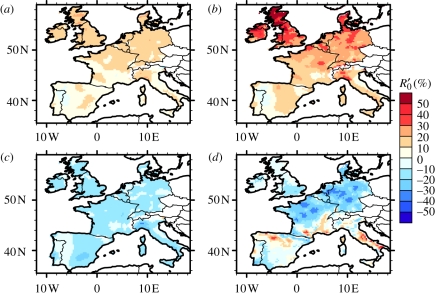
Sensitivity of *R*_0_ parameters to climate change. *R*_0_ relative anomalies (%) for the period 2000–2008 with respect to the 1961–1999 climatology (ASO) based on the climate observations. All parameters are assumed to be constant except one in each panel: in (*a*) the biting rate; in (*b*) 1/mean extrinsic incubation period; in (*c*) the vector mortality rate; and in (*d*) the vector-to-host ratio (*m*).

In NW Europe, these positive *R*_0_ anomalies are linked to changes in the biting rate (*a*) (figure 3*a*), and particularly the extrinsic incubation period (*ν*) (figure 3*b*). By contrast, changes in the vector mortality rate and *Culicoides* density do not explain this increase (figure 3*c*,*d*). In SW Europe, the influence of changing biting rate and extrinsic incubation period on the *R*_0_ anomaly is reduced compared with the NW, but there is a substantial contribution from changes in the vector-to-host ratio in parts of Spain, France and Italy, related to spread in the distribution of *C. imicola* (figure 3*d*).

**Figure 4.**
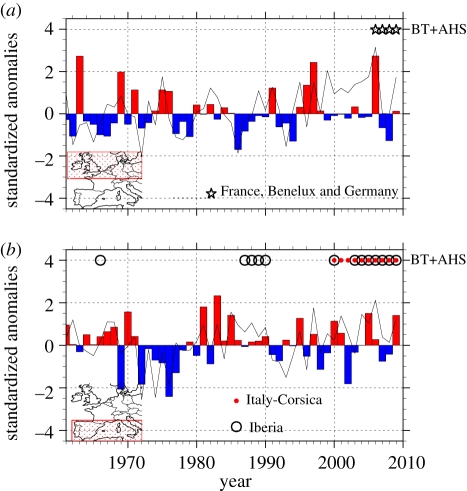
*R*_0_ recent evolution. (*a*) *R*_0_ standardized anomalies (histogram) over northwest and (*b*) southwest Europe. The standardized anomalies are computed retrieving the long-term mean and then weighting by the standard deviation (ASO 1961–1999). The area to compute the indices is displayed in the lower left corner. The observed BT and AHS outbreaks are represented for different areas by the associated markers. *R*_0_ is estimated from the observed climate dataset. The line represents anomalies of 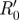 owing to the variations of the three virus transmission parameters (*a*, *μ* and *ν*) with the vector-to-host ratio, *m*, held constant.

The first ever occurrence of BT in NW Europe [43] occurred in the year for which the model has the largest positive anomaly since 1961 (figure 4*a*, histogram). The origin of the viral introduction in 2006 is still not known; however, our model shows that climate rendered that year in northern Europe at higher risk of a BT outbreak (in the event of viral introduction) than any of the previous 45 years. The fact that anomalies are negative for 2007 and 2008 in the NW, while the epizootic continued to spread, is not necessarily a discrepancy as *R*_0_ estimates only the initial spread of a disease, and in 2007 (and 2008), there were already significant numbers of infected holdings from which the disease disseminated. Other years of relatively high risk were 1963 and the mid-1990s. In SW Europe (figure 4*b*), there is a higher proportion of years with positive anomalies than in NW Europe, although they are generally of smaller magnitude. No BT was detected in SW Europe between 1961 and 1999. Nevertheless, there were outbreaks of a closely related viral disease of equids, African horse sickness (AHS), also transmitted by *C. imicola*, in Iberia in 1966 and in 1987–1991, both periods of positive anomaly for BT risk. The only decade without any recorded activity of either BT or AHS was the 1970s, a decade of consistently high negative *R*_0_ anomaly. Finally, considering BT in SW Europe in the last decade, the year with the strongest negative anomaly, 2002, stands out as the only year without new serotype introduction, and an unusually low reported BT incidence (see OIE Handistatus II Annual animal disease status Europe/2002/Bluetongue Animal health status at http://www.oie.int/hs2/sit_mald_cont.asp?c_mald=10&c_cont=4&annee=2002).

In figure 4*a*,*b*, the line represents the anomalies of 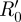 owing to the variations of the three virus transmission parameters (i.e. *a*, *μ* and *ν*) with the vector-to-host ratio, *m*, held constant. It shows that in NW Europe, over the last 10 years, 2006 stands out as the year when both *m* and the other transmission parameters were favourable for transmission. Conversely, between 2000 and 2005, all the transmission parameters but *m* were favourable. In SW Europe, the anomalies of 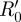 are more concordant than those of *R*_0_ with the epizootics of AHS which occurred between 1987 and 1990 on the Iberian peninsula, and with the epizootics of BT in 2004 and 2006 in southern Europe. This suggests that the climate in those years may have favoured disease transmission because of effects on the ability of the vectors to transmit the causative virus, rather than effects on the vector population size.

### 3.3. Future *R*_0_ anomalies

**Figure 5.**
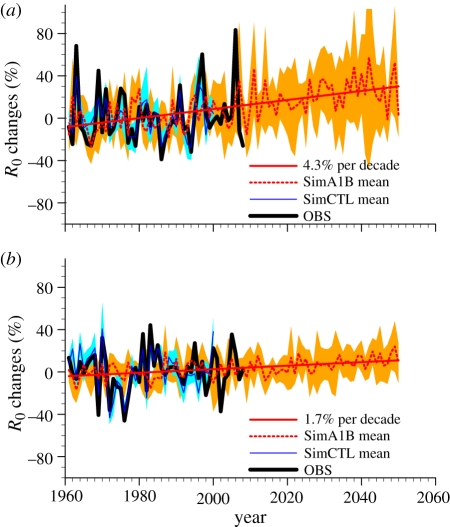
Simulated *R*_0_ future evolution. *R*_0_ relative anomalies (%) with respect to 1961–1999 time period in ASO for (*a*) northwest and (*b*) southwest Europe (see figure 4 for the domain definition). *R*_0_ estimated from the climate observations (OBS) is displayed in black, the *R*_0_ ensemble mean based on the SimCTL (SimA1B) RCM ensemble is displayed in blue (red). The blue (orange) envelope highlights the spread (defined as 1 s.d. of each RCM realization to the ensemble mean) within the SimCTL (SimA1B) ensemble.

The outputs from SimCTL (the ensemble of simulations fitted to past and present climatic conditions) integrated within the BT model reproduce very well the variability in *R*_0_ for the past period (1960–2000), in both NW (figure 5*a*) and SW (figure 5*b*) Europe. This confirms that simulated climate data can successfully drive the integrated BT model. Integrating SimA1B (the ensemble of simulations run under the climate change special report on emission scenario A1B), the mean *R*_0_ across the 11 RCMs is simulated to increase gradually between the present and 2050, but more rapidly in NW than in SW Europe (4.3% versus 1.7% per decade). Model outputs also exhibit greater spread across the different RCM projections in the NW. Given susceptible ruminant host populations, our models suggest that by 2050, *R*_0_ will have increased by 30 per cent in NW and 10 per cent in SW Europe, with respect to 1961–1999 mean modelled risk levels in each of the two regions. Nevertheless, even in 2050, the absolute risk of BT transmission remains twice as high in SW than in NW Europe.

### 3.4. Influences of temperature and rainfall on *R*_0_ anomalies

**Figure 6.**
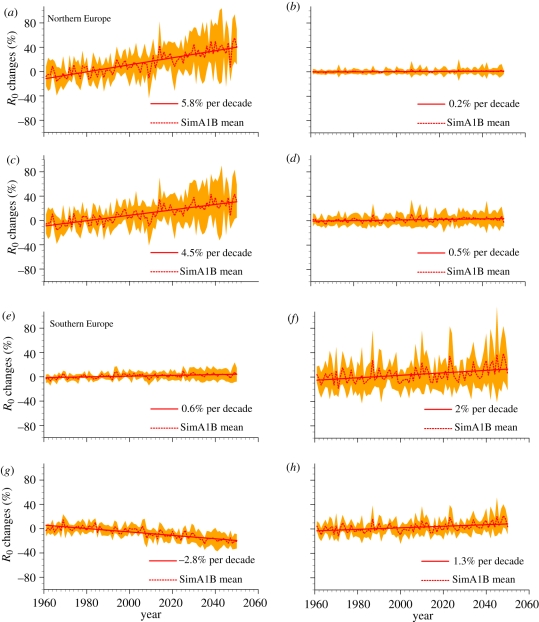
Sensitivity of *R*_0_ to changes in rainfall and precipitation. *R*_0_ relative anomalies (%) with respect to 1961–1999 time period in ASO for (*a*–*d*) northwest and (*e*–*h*) southwest Europe (see figure 4 for the domain definition). The anomalies are computed fixing (*a*,*e*) either precipitation constant to 500 mm or (*c*,*g*) to 250 mm or (*b*,*f*) temperature constant to 25°C or (*d*,*h*) to 20°C.

For NW Europe (figure 6*a*–*d*), *R*_0_ anomalies are mainly driven by changes in temperature, particularly via its influence on extrinsic incubation period, and, to a lesser extent, the biting rate. By contrast, with temperature set constant, there is very little trend in future *R*_0_ anomaly (less than 0.5% change per decade).

For SW Europe, the results are less robust (figure 6*e*–*h*). When temperature is set constant and only rainfall varies (figure 6*f*,*h*), the increase in *R*_0_ anomaly is 2 or 1.3 per cent per decade (20°C and 25°C, respectively), mainly via the effect of rainfall on vector-to-host ratio (*m*). However, the relation is complex, leading to opposite trends in *R*_0_ anomaly when rainfall is set constant (figure 6*e*,*g*). When rainfall is set at low values (*p* = 250 mm), the increase in temperature causes decrease in *R*_0_ anomaly while, at higher rainfall levels, increasing temperature leads to a slight increase in *R*_0_ anomaly. In other words, if SW Europe is dry, the vector-to-host ratio is simulated to decrease as temperatures increase, leading to the decrease in BT transmission risk. Conversely, if SW Europe is wetter, the increase in temperature then leads to a moderate increase in *R*_0_.

### 3.5. Evaluation of the uncertainty of future *R*_0_ anomalies

**Figure 7.**
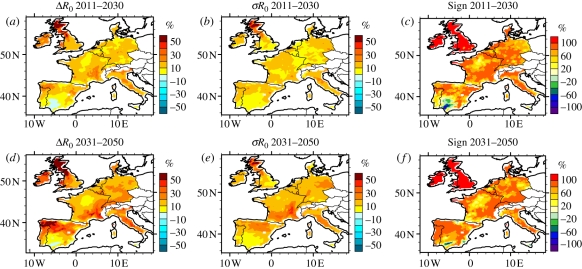
Simulated *R*_0_ changes, spread and sign consistency. (*a*,*d*) Simulated mean *R*_0_ changes (%) estimated from the SimA1B ensemble (with respect to the 1961–1999 climatology). (*b*,*e*) Multi-model spread defined as 1 s.d. of all RCM projections with respect to the ensemble mean. (*c*,*f*) Sign consistency (%), if all RCM projections agree on an increase, then the value is 100% and conversely for a decrease, −100%.

Focusing on the effects of future regional changes of climate (figure 7), *R*_0_ is simulated to increase by the SimA1B RCM ensemble over most of Western Europe. For the 2011–2030 period, climatic changes are projected to induce a significant increase in *R*_0_ in Ireland, Wales, southeast France and NW Iberia (figure 7*a*) consistent between the different climate models in terms of direction (figure 7*c*), and with a moderate spread in the projected magnitude (figure 7*b*). For the 2031–2050 period, Ireland, a larger part of Britain and NW Iberia show similar patterns (figure 7*d*–*f*). Over that period, changes in climate also induce a significant increase in BT risk in southeast France, but with a large spread in magnitude (figure 7*e*). By contrast, a limited area of southern Spain exhibits a small decrease in *R*_0_ which is consistent between models and in both time periods.

## 4. Discussion

The dramatic emergence of BT in Europe—a disease with a known strong dependence on climate [44]—has enabled testing of our novel and generic framework to assess the effects of climate on the transmission of a disease. Applied to BT, the framework gives the best evidence to date that BT's emergence across Europe is related, at least partly, to climate change. It enabled the assessment of the changing risk of BT transmission (in the event of an introduction of the pathogen) in space and time, and also in which climate-sensitive biological mechanisms were involved. It further allowed us to investigate the change in risk of disease transmission in the future, given climate change, and to assess the relative influences of temperature and rainfall on this change in disease risk. This type of framework can be applied to other diseases for which the epidemiology is well described and where the links between the *R*_0_ parameters and climatic variables have been quantified.

The main limitations of the application of this framework to BT are owing to paucity of species-specific entomological knowledge on the parameters that determine vectorial capacity of European vectors. The complete list and relative importance (including competence) of the *Culicoides* species involved as well as their distribution and fine estimates of their biological parameters (such as the biting rate, extrinsic incubation period (EIP), mortality rate) and of the vector-to-host ratio would enable more robust modelling.

For example, the fact that the densities of *C. imicola* are overestimated in northern Italy could show that we omitted a factor influencing the establishment of this species. Conte *et al*. [27,28] have shown that soil type and vegetation cover impacted on its distribution in Italy. This information could be used to refine the distribution models. This can be done quite easily for variables which do not vary in time such as the soil type (supposing the data were available for the whole study area), but remains more difficult for other environmental variables that may be important, such as forest cover, which has varied over the last 50 years in Europe.

Modelling of the vector-to-host ratio component of the *R*_0_ disease model is problematic. First, the *Culicoides* population caught in light traps may not be fully representative of those that transmit BT virus. In one study, CDC (Center for Disease Control) traps baited with UV light tended to overestimate the numbers of *C. imicola* vacuumed off a sheep while underestimating those of *C. obsoletus* (although only 11 *C. imicola* were caught during the 8 days of the study [45]). Biteau-Coroller [46] captured once 23 per cent more and once 26 per cent less *C. imicola* in an Onderstepoort Veterinary Institute (OVI) trap than in a drop trap. Carpenter *et al*. [47] suggest that OVI light traps underestimate the role of some potential vector species such as *Culicoides chiopterus*. On the whole, while it is likely that light trap catches do not fully represent the population that feed on hosts, there is still no consensual method to correct for the bias. A second problem is that it remains unclear whether the number of midges caught in a trap should be treated as an estimate of the vector population size (as did Gubbins *et al.* [8] and Hartemink *et al.* [9]) or the vector-to-host ratio (as assumed here). We consider there to be problems with the former approach. First, there is no information on how to relate the number of midges caught in a trap to the number in the region being modelled: [9], for example, multiplied by an arbitrarily chosen constant (100) to convert a trap catch to the midge population per square-kilometre. Secondly, and as described earlier, it leads to problematically extreme estimates of *R*_0_ at extreme host densities (high or low). We consider that our novel approach, which uses trap catch as an estimate of the vector-to-host ratio, has the advantage of bypassing the problems outlined above and ensures that *R*_0_ is proportional to trap catch. Nevertheless, formal testing of the two hypotheses has not yet been undertaken.

The observation that in southern Europe annual anomalies of 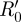 (i.e. *R*_0_ modelled with varying viral transmission parameters but constant vector-to-host ratio) tended to be more concordant with epizootics than anomalies of *R*_0_ itself (with vector-to-host ratio also varying) is interesting. While there are insufficient data to draw robust conclusions, one possibility relates to the absence of lags in our model. Our model presumes near-instantaneous effects of climate variation on *R*_0_ (at least within the same three month period). In reality, the viral transmission parameters (*a*, *μ* and *ν*) may be expected to respond quite rapidly to changes in climate, but there are likely to be significant lags in the response of the vector-to-host ratio, while the population size builds up and/or spreads over the years. If correct, this effect would probably affect the utility of annual anomalies in *R*_0_ (but not 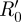), but should affect decadal anomalies in *R*_0_ less.

Despite these weaknesses, our novel framework successfully describes many aspects of BT's emergence, as demonstrated by the good concordance between model outputs and the observed distribution of the vectors and the disease. This shows that these potential biases have only had a moderate impact on the analysis. Nevertheless, given these approximations, we recommend not to compute absolute values of *R*_0_ but, until further data are available, to focus analysis on trends and anomalies.

Another limit of the application is that we explored only the effects of changing climate over time on BT's *R*_0_, holding constant other factors which may also vary in time such as host densities. The fact that variation in climate alone successfully reproduces many aspects of this past emergence does not mean that climate is the only driver, but it provides strong evidence that climate has played an important role. Indeed, ignoring a major driver would most likely result in substantial spatial and temporal discrepancies between predictions and the observed situation.

Further, this framework provides mechanistic insight into the drivers of the emergence, highlighting for the first time the role of climate-drivers of virus transmission, particularly the extrinsic incubation period, in NW Europe and a relatively larger role of climate-drivers of vector densities in SW Europe. It also confirms the role of temperature as a major driver of change in NW Europe (through the changes in EIP it produces), and a more complex situation in SW Europe where temperature influences differently the vector-to-host ratio and therefore *R*_0_ depending on whether the area is drier or wetter. When rainfall is low, the increase in temperature will lead to a decrease in risk, whereas when it is high, the increase of temperature will lead to an increase in risk. Finally, it has also permitted quantification of the effects of future changes of climate on BT risk of transmission in the event of viral introduction, projecting the *R*_0_ for BT to increase still further across much of Western Europe over the next 40 years, indicating an increasing threat to susceptible livestock from this disease.

Future simulations of the effects of climate on the disease must be interpreted with caution as there are still uncertainties related to the actual state-of-the-art climate model biases and associated with the selected emission scenario (A1B). Such approaches can be updated on a continuous-flow basis when climate data emanating from new models and/or forced by new emission scenarios are made available. Further research should also focus on testing the effects of a wider range of climatic variables on transmission parameters, including other variable statistics which could be more pertinent than mean values such as cumulative (e.g. degree days) or fluctuation [48] functions and evaluating the uncertainty of these relations. In parallel, climatic models and simulations need to be developed for these variables.

We have assessed only the effects of climate (mainly temperature and precipitation) on future *R*_0_ via direct effects on vectors and viral transmission parameters. This approach, namely holding constant non-climatic drivers, corresponds to the first of three contexts in which the effects of climate change on health can be evaluated [49]. The other contexts require the inclusion of non-climatic disease drivers in models. For BT, substantial knowledge gaps would need to be filled in order to take into account both the indirect knock-on effects of climate change [49] (on social, economic, political and land-use [50] changes), and non-climatic disease drivers. Although other possible non-climatic drivers of past BT occurrence have been discounted (see electronic supplementary material) [3]; for future periods, non-climatic drivers such as changes in livestock densities (or composition), and the development of novel control tools which reduce the exposure of naive hosts to vectors should not be ruled out as they could have major impacts on the future occurrence of BT. Indeed, we cannot be sure that other disease drivers will not arise and counteract or supplant these effects in the future.

The real future of BT in Europe will result from the combination of both climatic and non-climatic future changes. Further development of our approach would be the inclusion of additional, non-climatic drivers of BT spread; this implies that spatio-temporal estimates of the drivers, and their future trends, are available, and that their link to disease transmission is quantified. The ultimate aim would be to disentangle the interactions between drivers in order to then apply this approach for all drivers combined. Much more knowledge is needed about the different BT episystems (vectors, hosts, pathogens, biological controlling mechanisms and all the environmental factors), and on their future trends, before one can hope to reach this point.

Finally, simulating the effects of future climate on the risk of transmission of a disease in the event of viral introduction is very different from predicting where and when a disease will occur. The consequence of this is that the validation of this type of model is complex. Indeed, a year with no epizootic does not mean that the conditions were not favourable for an epizootic; it might just be that no pathogen was introduced or that animals had been vaccinated. Thus, we can only verify the sensitivity of the model by comparing the first year of emergence of a new epizootic with the model's projections. Predicting where and when a disease will occur is out of the scope of the framework proposed here and, perhaps, remains a problem too complex to be addressed [51] as there may be changes over time in hosts, vectors, pathogens and the environment (including climate) and, most importantly, as this also depends on the probability of introduction of the pathogen.
